# Supplementary material for: The Evolution and Characterization of the RNA Interference Pathways in Lophotrochozoa
Source: Genome Biol Evol. 2024 May 7;16(5):evae098. doi: 10.1093/gbe/evae098 (PMC11114477; doi:10.1093/gbe/evae098)
Supplement: evae098_Supplementary_Data [file evae098_supplementary_data.zip › Supplementary_materials.zip!/selected_miRNAs/CrGi_selected_miRNAs/CrGi_NC_047563.1_16214.pdf]

Provisional ID	:	NC_047563.1_16214
Score total	:	88.1
Score for star read(s)	:	3.9
Score for read counts	:	81.7
Score for mfe	:	1.5
Score for randfold	:	1.6
Score for cons. seed	:	-0.6
Total read count	:	172
Mature read count	:	165
Loop read count	:	0
Star read count	:	7

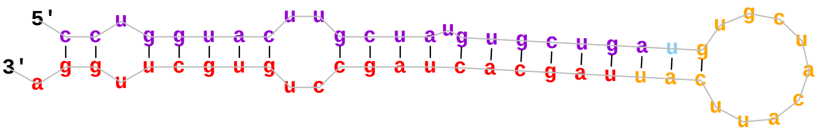

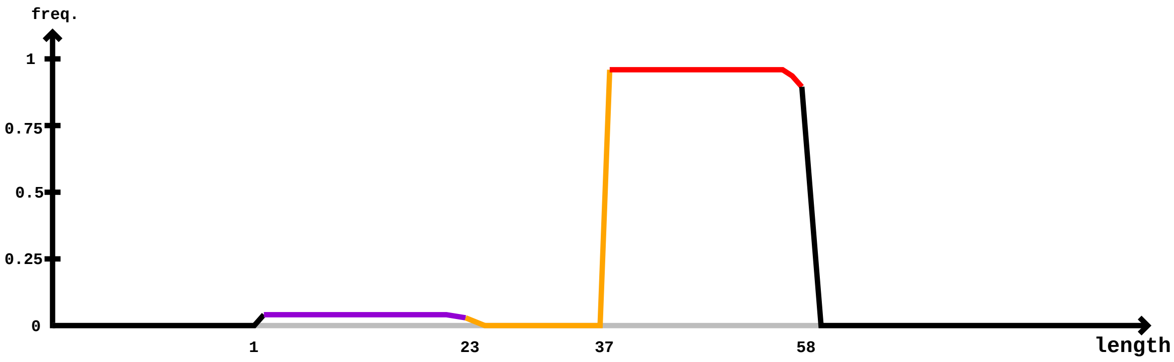

Star **Mature**

```
5'- uaaaucaagacuuguaauuuccugguacuugcuaugugcugaugugcuacauucauuagcacuagccugugcuuggagacaaaggucugaaaaucaacuacauguucauguu  -3'  obs
    uaaaucaagacuuguaauuuccugguacuugcuaugugcugaugugcuacauucauuagcacuagccugugcuuggagacaaaggucugaaaaucaacuacauguucauguu       exp
    ....(((.(((((....(((((.(((((..((((.(((((((((.........)))))))))))))..))))).)))))...)))))))).........((((....)))).   reads   mm   sample
    ........................................................uagcacuagccugugcuGg.....................................   1       1    Ov2
    ........................................................uagcacuagccugugcuGgga...................................   28      1    Ov2
    ........................................................uagcacuagccugugcuGggag.................................   5       1    Ov2

    ....................ccugguacuugcuaugugcuga......................................................................   1       0    Ov1
    ........................................................uagcacuagccugugcuGgga...................................   26      1    Ov1
    ........................................................uagcacuagccugugcuGggag.................................   2       1    Ov1

    ....................ccugguacuugcuaugugcug.......................................................................   1       0    Mt1
    ....................ccugguacuugcuaugugcuga......................................................................   2       0    Mt1
    ........................................................uagcacuagccugugcuGg.....................................   1       1    Mt1
    ........................................................uagcacuagccugugcuGgg....................................   2       1    Mt1
    ........................................................uagUacuagccugugcuugga...................................   1       1    Mt1
    ........................................................uagcacuagccugugcuGgga...................................   35      1    Mt1
    ........................................................uagcacuagccugugcuGggag.................................   11      1    Mt1

    ....................ccugguacuugcuaugugcu........................................................................   1       0    Mt2
    ....................ccugguacuugcuaugugcuga......................................................................   2       0    Mt2
    ........................................................uagcacuagccugugcuGg.....................................   2       1    Mt2
    ........................................................uagcacuagccugugcuGgg....................................   5       1    Mt2
    ........................................................uagcacuagccugugcuGgga...................................   42      1    Mt2
    ........................................................uagcacuagccugugcuGggag.................................   4       1    Mt2
```
